# Perioperative Management of Patients Undergoing Periacetabular Osteotomy: An Evidence-Based Review

**DOI:** 10.1007/s12178-026-10049-4

**Published:** 2026-06-27

**Authors:** Conor Dolson, Ira Zaltz, Alex L. Gornitzky

**Affiliations:** 1 Department of Orthopaedic Surgery, Corewell Health William, Beaumont University Hospital, Royal Oak, MI USA; 2 Department of Orthopaedics, C.S. Mott Children’s Hospital, University of Michigan, 1540 E. Hospital Drive, SPC 4241, Ann Arbor, MI USA

**Keywords:** Periacetabular osteotomy, Perioperative, Mental health, Preoperative, Rehabilitation, Review

## Abstract

**Purpose of Review:**

Periacetabular osteotomy (PAO) is an established and effective surgical treatment for symptomatic acetabular dysplasia in adolescents and young adults with healthy articular cartilage. While outcomes and technical surgical considerations are well described, perioperative management practices vary significantly across institutions. The purpose of this narrative review was to synthesize current literature on evidence-based best practices for perioperative management of patients undergoing PAO and to identify knowledge gaps for future investigation.

**Recent Findings:**

A comprehensive review of the literature was performed focusing on perioperative care across four phases: preoperative, intraoperative, acute postoperative, and rehabilitation. Areas of emphasis included laboratory evaluation and nutritional optimization, mental health screening, patient education, blood loss mitigation strategies, pain control, venous thromboembolism and heterotopic ossification (HO) prophylaxis, and postoperative rehabilitation protocols. Strong evidence exists to support specific intraoperative practices such as tranexamic acid administration, controlled hypotension, and fascia iliacus blocks for pain control. Conversely, limited or heterogeneous evidence exists regarding preoperative laboratory testing, psychosocial interventions, standardized opioid prescribing, venous thromboembolism prophylaxis regimens, and rehabilitation protocols.

**Summary:**

Psychosocial health and preoperative educational interventions appear underutilized despite growing evidence of their impact on outcomes. Significant gaps remain, particularly regarding preoperative optimization and rehabilitation strategies. Continued high-quality research is needed to establish standardized, evidence-based perioperative care pathways to further improve outcomes following PAO.

## Introduction

The Ganz periacetabular osteotomy is an effective treatment for patients with symptomatic hip dysplasia who have healthy articular cartilage [1]. Large cohort studies have shown favorable short- and long-term patient-reported outcomes as well as low complication rates [2–4]. Furthermore, PAO alters the natural history of dysplasia, delaying or preventing the need for arthroplasty [5].

Numerous risk factors have been shown to modulate outcomes of PAO and hip preservation surgery, including operative technique [6, 7], demographic factors [2], adherence to protocols and rehabilitation [3, 8], and more recently, psychological and behavioral factors [9–12]. While recent work has sought to quantify and describe optimal surgical technique [13–15], perioperative management is inconsistent between centers. A recent survey of PAO surgeons in the ANCHOR study group revealed “significant practice variability in peri-operative pain management, anesthesia, physical therapy, and VTE and HO prophylaxis” [16], highlighting opportunities to improve patient care with evidence-based practice. In addition, there has been renewed understanding of the impact of patient’s expectations and psychosocial health on post-surgical outcomes, with some studies showing a greater impact of these factors on patient-reported outcomes than surgical technique [9, 17, 18].

The purpose of this study was to comprehensively review the literature for perioperative management surrounding the PAO in otherwise healthy adolescents and young adults. Our secondary goal was to identify opportunities for further research to optimize perioperative PAO care pathways.

## Methods

Literature was identified using a Pubmed search of all topics related to perioperative management of PAO plus a hand-search of references in all identified literature. Where possible, articles focusing on otherwise healthy adolescents and young adults with idiopathic hip dysplasia were selected, and articles describing management in patients with neuromuscular or non-idiopathic causes were excluded. Emphasis was placed on stronger levels of evidence published within the past five years (2021–2026). Areas of limited evidence were identified and discussed. Finally, perioperative management was divided into four phases according to chronological relationship to surgery: preoperative, intraoperative, acute postoperative (first 6-weeks following surgery), and rehabilitation (from 6-weeks postoperative onward).

### Management Through the Phases of Perioperative Care

#### Preoperative Phase

The foundation of hip dysplasia care includes a complete history and physical examination, comprehensive imaging evaluation (both radiographs and advanced imaging), preoperative laboratory evaluation (as appropriate), patient education and expectation setting, and mental health screening and referral (as appropriate). Non-idiopathic etiologies of dysplasia must be ruled out. Additionally, evaluating for concurrent pathology in the proximal femur and/or intra-articular space is important. Finally, perioperative psychosocial preparation of the patients and their support system is a valuable opportunity to streamline care and improve PROs [8].

For any adolescent or young adult patient presenting with hip pain, an in-depth history and physical exam are required both for proper diagnosis and to rule out other potential diagnoses. Hip dysplasia symptoms commonly present as insidious-onset pain in a “C” shape around the hip from the inguinal crease toward the buttock that may worsen with activity, as well as extended sitting or standing [19]. High-level sports participation is common. Prolonged activity can lead to abductor fatigue with associated weakness, causing lateral hip pain [20]. Patients may also report mechanical symptoms, such as clicking and popping, which can occur due to iliopsoas snapping over the exposed anterior femoral head, synovitis, or intraarticular chondral pathology. While factors such as female sex, family history, firstborn, and intrauterine breech position are associated with neonatal hip dysplasia, previous work has suggested that patients diagnosed with dysplasia later in life likely would not have met American guidelines for ultrasound screening and had normal examinations as infants [21].

On exam, increased hip range of motion (ROM) may be associated with instability. This typically presents as increased ROM in all directions, particularly rotation in flexion, due to a combination of capsular laxity and generalized hypermobility, deficient bony containment, and commonly associated increased femoral anteversion. Gait examination (Trendelenburg gait) and abductor strength assessment is important, as the hip abductors (gluteus medius and minimus) are the primary dynamic stabilizers of the pelvis during single-leg stance. As a result, understanding baseline abduction strength is important for identifying opportunities for improved rehabilitation/strengthening, identifying concomitant pathology (e.g. femoral anteversion), and providing a baseline to assess future clinical outcomes [22]. Global ligamentous laxity that may exacerbate instability should be assessed clinically using Beighton’s scoring system. Numerous special tests with varying degrees of sensitivity and specificity have been devised to test for hip instability, including the prone apprehension relocation test (PART), prone external rotation test, anterior and posterior apprehension tests, lateral hyperextension-abduction-external rotation test and axial distraction tests. Descriptions of these techniques and their reliability can be found in the published literature [22, 23]. Other sources of hip pain should be evaluated as well, with some of the more frequent confounding diagnoses in the PAO population including, but not limited to, neurologic disorders, radicular pain, femoroacetabular impingement, coxa saltans, Perthes disease, slipped capital femoral epiphysis, tendonitis, apophysitis, synovitis, inflammatory disease, early arthritis, and stress fracture [22]. Spinal and abdominal pathology must be ruled out as well. There are many published comprehensive descriptions of the pre-operative history and physical exam for hip dysplasia and discussion is beyond the scope of this review.

#### Imaging

Strong evidence supports the use of radiographs and computed tomography (CT) for diagnosis of DDH and preoperative planning. Standard radiographic series include anterior-posterior (AP), false profile, and Dunn lateral views. Standing radiographs may better represent the patient’s functional pelvic position during weightbearing, and have been shown to correlate closely with CT measurement [24]; however, some centers prefer supine radiographs for standardization and easier comparison with advanced imaging. A multitude of radiographic measurements and signs have been reported, such as lateral center edge angle (LCEA), anterior center edge angle (ACEA), Tonnis angle, femoroepiphyseal acetabular roof (FEAR) index, anterior and posterior wall indices, alpha angle, crossover sign, or the windshield wiper sign [13, 19, 20, 25–27]. Measurements may differ significantly depending on position, thus care should be taken when comparing office standing radiographs and supine intraoperative radiographs [28]. Published data on the cutoff values and reliability of these measurements varies widely. Computed tomography (CT) is particularly useful in evaluating acetabular and femoral 3-dimensional anatomy, including version measurements of both structures [25]. It is also used to calculate anterior, posterior, and horizontal acetabular sector angles, which assist in understanding the specific regions of acetabular deficiency and inform ideal intraoperative correction [29].

MRI as well as contrast-enhanced MR arthrography (MRA) is used to evaluate cartilage or labral pathology, such as cartilage wear and arthritis, cartilage delamination, or labral anatomy potentially identifying patients with advanced cartilage wear who may not be candidates for PAO [13, 30]. The significance of labral findings in the context of hip dysplasia remains controversial, however, with recent studies demonstrating limited clinical benefit at one-year follow-up of simultaneously performing hip arthroscopy at the time of PAO [31].

Dynamic ultrasound has received renewed attention due to its benefit as a radiation-free imaging tool with low cost, but utility remains technician- and resource-dependent. Dynamic ultrasound can identify hip joint microinstability by measuring translation of the femoral head relative to the acetabulum while the hip is passively manipulated or during an apprehension test, offering significantly more information on the function and stability of the joint compared to static imaging modalities [32, 33]. Recent results have shown strong inter-rater and intra-rater validity of ultrasound exams for dysplasia, but sensitivity and specificity of findings remains to be verified [22, 34, 35]. Further evidence is needed to clearly establish the diagnostic role of ultrasound.

#### Laboratory Evaluations

There is currently no standardized laboratory workup prior to PAO, and protocols across large academic medical centers vary significantly [36]. Current practice is extrapolated from patient blood management literature and general perioperative anesthesia guidelines for high blood‑loss orthopedic surgery [37].

In practice, preoperative evaluation may include complete blood count and metabolic profile to screen for abnormalities. Iron deficiency anemia affects up to 40% of pediatric patients [38], and blood loss during PAO can vary from 100 to 3900 mL, with mean blood loss typically around 700mL [3, 39]. Thus, preoperative identification of anemia can allow for supplementation and prevention of postoperative anemia or other complications. Level 4 evidence from adult spine surgery has shown decreased complications in patients taking preoperative iron supplementation [40]. Vitamin D levels may also be assessed preoperatively, as low levels have been shown to increase risk of delayed union and stress fracture in PAO patients [41].

Alternatively, many preoperative protocols include empiric pre-operative supplementation with Vitamin D, calcium and iron regardless of pre-operative levels, thus obviating the need for additional laboratory evaluation [36, 42]. Vitamin C can be considered to help increase iron absorption [43]. Other trace minerals, such as selenium, copper, and zinc, also play an important role in bone metabolism, and there is evidence that supplementation of these minerals (with a generic multivitamin) can improve overall bone health and healing [44, 45].

Other biomarkers to predict healing following PAO have not yet been identified, though level 3 evidence has shown risk factors for nonunion after PAO to include lower bone mineral density and higher BMI [46]. Nicotine has been repeatedly identified as a risk factor for nonunion and delayed union in the general orthopedic literature [47]. Additionally, marijuana exposure may increase time to union in extremity fractures in pediatric patients [48]. Increasing rates of use suggest that screening may be appropriate in at-risk patients.

#### Mental Health Evaluation

Recent studies in PAO patients have sought to understand the interconnection between pain, psychiatric symptoms, and post-surgical outcomes. Depression and anxiety have been shown to negatively affect both preoperative and postoperative outcomes in patients undergoing PAO and hip arthroscopy [49], with one study concluding that mental health status was the sole predictor of postoperative pain and patient-reported outcomes following PAO [50]. Specifically, depressive symptoms or pain catastrophizing behaviors can significantly alter patients’ postoperative course and overall function [18]. Psychosocial symptoms are more prevalent in adolescent patients with chronic hip pain compared to the general population, so proper pre-operative identification, referral, and treatment as appropriate may improve outcomes [10]. In a prospective study, Richard et al. demonstrated improvements in self-reported psychological metrics after PAO when patients were concurrently treated by a staff psychologist [8]. Furthermore, despite identifying only a limited number of studies, a 2025 review highlighted improvements in psychological well-being as well as physical functioning for patients undergoing psychological interventions [51], As surgeons are often not trained or experienced in this care, holistic inter-disciplinary collaboration between surgeons, primary care physicians and mental health professionals is critical to improving outcomes [17]. Synthesizing the literature, the Layer+ model seeks to account for the impact of psychosocial factors on outcomes, and suggests that multidisciplinary efforts to improve outcomes are a crucial part of perioperative management for hip preservation patients [9]. Further work is necessary to determine who might benefit from various types of psychological interventions.

#### Education and Expectations

Expectations matter. In a survey of experienced PAO surgeons, nearly half reported that surgeon and patient expectations do not align “most of the time”, with patients expecting complete resolution of pain and unrestricted return to desired activities while surgeons base success on pain reduction and functional activities [52]. Analysis of social media has helped reveal patient concerns preoperatively and postoperatively, with many asking questions about preoperative education, rehabilitation, complications, and chronic pain [53]. Educational interventions have been shown to safely and effectively reduce pain and medication usage, improve patient mental health symptoms, especially anxiety, and improve patient satisfaction. A 2023 narrative review examining preoperative educational interventions across a variety of surgical specialties demonstrated that nearly all educational interventions were effective to some degree, with particular emphasis on decreasing opioid consumption following preoperative education [54]. Further work is needed to explore what types of perioperative education and expectation-setting interventions are effective at improving outcomes, including when, how and by whom they should be delivered.

### Intraoperative Phase

#### Approach

The surgical approach to the PAO has evolved over the years, with the original description by Ganz exposing the ilium medially and laterally. Protecting the abductors in the current approach preserves both muscle function and acetabular blood supply [55]. Proper management of the soft tissues also limits the risk of HO and facilitates acetabular reorientation [7]. Other approaches, such as the transtrochanteric approach, may increase risk of osteotomy nonunion [41]. The effects of variations in surgical skill level have not been effectively studied.

#### Blood Loss

Osteotomy of the highly vascularized bony pelvis can lead to significant blood loss [56]. Allogenic blood transfusion carries risk of adverse reactions, infection, and greater surgical cost. Techniques to minimize blood loss, including hemodynamic control, tranexamic acid (TXA) administration, and autologous red blood cell donation are intended to minimize acute blood loss and transfusion rates during/following PAO.

Controlled intraoperative hypotension is an established technique to reduce blood loss and improve surgical efficacy through improvement in surgical visualization. Multiple systematic reviews, including those specific to pediatric patients, have shown the pressure-dependent effectiveness of this technique in reducing blood loss (i.e. lower mean arterial pressure (MAP) correlated with lower blood loss) and in reducing need for transfusion [57]. Despite its established track record, existing evidence is underpowered to ensure this technique’s overall safety, necessitating close anesthesia monitoring during use [58]. The authors’ preference is to maintain a MAP of 60 mm Hg throughout PAO surgery.

Strong evidence exists for the safety and efficacy of TXA in preventing excessive blood loss in many orthopedic surgeries, and specifically during PAO surgery. In a double-blinded randomized controlled trial of 81 PAO surgeries, patients receiving 2 weight-based doses (maximum one gram) of intravenous TXA, one at time of incision and one at time of closure, demonstrated significantly lower mean calculated blood loss and allogenic transfusion rates than patients receiving 2 doses of normal saline, with no additional venous thromboembolisms (VTEs) identified [59]. Another retrospective study using similar dosing of two 1 g infusions of TXA found similar results of decreased blood loss without increased risk of VTE in a series of 100 patients [60]. A similarly structured retrospective review of 96 patients using 10 mg/kg/h continuous dosing of TXA again found lower blood loss and transfusion risk without increased VTE risk [61]. A 2016 study of 137 patients undergoing PAO reported a 0% rate of allogenic transfusion in the tranexamic acid group versus 21% in the control group, with no significant increase in VTE risk [62]. Finally, a systematic review of five studies involving 507 patients concluded that the administration of antifibrinolytics, including TXA, significantly reduced the total estimated blood loss and allogenic transfusion rate in PAO, though there was insufficient data to calculate venous thromboembolism risk [56]. Altogether, the evidence strongly supports the use of TXA during PAO surgery.

Autologous blood donation using intraoperative cell salvage can reduce allogenic transfusion rates, and has been shown to be cost effective [63]. A 2023 systematic review of cell salvage in adults across a variety of surgical specialties found low-certainty evidence to support cell salvage to reduce transfusion rates in most types of orthopedic surgeries, including hip procedures, with no significant difference in rates of adverse events [64].

Preoperative autologous red cell donation has also been used to minimize allogenic transfusion; however, this practice has fallen out of favor due to concerns over preoperative lowering of hematocrit, higher overall transfusion rates (despite lower allogenic transfusion rates), wasted blood units, higher cost, and lack of benefit [65, 66],

#### Pain Management

Multiple intraoperative techniques can be used to improve post-operative pain. Strong evidence supports use of perioperative fascia iliacus blocks to improve postoperative outcomes. Multiple studies have demonstrated that compared to epidural analgesia, the fascia iliacus block allows patients to achieve PT goals more quickly, use less opioid medication, and shorten their length of stay, with one cohort study showing an average length of stay 1.5 days shorter with fascia iliacus block compared to epidural analgesia [67, 68].

Additional techniques borrowed from arthroplasty surgery may also hold promise. Liposomal bupivacaine and the “joint cocktail” of multiple medications injected into soft tissues surrounding the incision are techniques to reduce postoperative pain currently used effectively in arthroplasty. At present there is no published literature on their use in PAO [69]. Liposomal bupivacaine is a method of encapsulation to increase release time of bupivacaine when used in neuraxial analgesia, and is currently approved for interscalene, popliteal sciatic, and adductor canal blocks with frequent use in arthroplasty procedures [70]. While studies in other procedures are mixed on its effectiveness and ability to reduce opioid usage, liposomal bupivacaine has been shown to be safe, and might be a method for increasing effectiveness and duration of the fascia iliacus block [71]. The “joint cocktail”, a combination injection of Ropivacaine, Ketorolac, clonidine, and epinephrine commonly administered at the conclusion of arthroplasty procedures, is another technique that holds promise for improving post-PAO pain control. The specific drugs and dosages may vary between patients and procedures. These injections have been shown to be safe when appropriately utilized, but have not been prospectively evaluated in PAO [71]. The authors’ preference is to supplement a pre-operative fascia iliaca block with a post-operative, surgeon-administered joint cocktail consisting of a weight appropriate dose of 20 mL 0.5% Ropivicaine, 1 mL 30 mg/mL Ketorolac, 0.15 ml Epinephrine 1 mg/ml, and 0.8 mL 100 mcg/ml Clonidine into the soft tissues around the hip, with particular focus on numbing the sciatic, femoral and obturator distributions.

### Acute Postoperative Phase

The acute postoperative phase consists of the inpatient period and the first six weeks following surgery. In this phase, pain control, DVT prophylaxis, HO prophylaxis, and rehabilitation make up the most important components of peri-operative management.

#### Pain Control

As discussed above, intraoperative pain control methods as well as preoperative education can help promote early mobilization and reduce opioid usage. Level II evidence supports post-operative, multimodal analgesic regimens for reducing pain, opioid use, and length of hospital stay while simultaneously improving recovery and decreasing opioid-related side-effects [69]. A systematic review evaluating opioid prescription patterns in pediatric orthopaedic patients found that reported utilization of opioids was often much lower than prescribed, with under 60% of prescribed medication being taken, highlighting opportunities to reduce prescription numbers [72]. Standardized prescribing practices have been proposed to reduce overall usage and overprescribing, with one quality improvement study evaluating prescribing patterns for pediatric patients, including PAOs, showing that a standardized protocol involving review of medical history, physical examination, “red flags”, pain type, and initiation of opioid-sparing interventions resulted in much lower opioid doses prescribed on average [73].

#### DVT Prophylaxis

Thromboembolic events, while rare, can lead to significant morbidity and even mortality. In a comprehensive, multicenter study on PAO complications from the ANCHOR group, there was a reported VTE rate of 1.5% in 205 hips, despite all patients receiving chemical DVT prophylaxis. All patients were successfully treated with anticoagulation, and none suffered long-term sequelae [3]. A 2016 retrospective study of 87 cases of PAO and 643 femoroacetabular osteotomies identified only 2 cases of VTE, with the majority of the patients in the study receiving aspirin 81 mg or 325 mg twice daily, concluding that aspirin is an adequate DVT prophylaxis in hip preservation surgery [74]. Similarly, Kraeutler et al. reported a 0% incidence of clinically apparent DVT when low-dose aspirin was combined with mechanical prophylaxis following 145 hip surgeries [75]. Likely owing to the risk involved and effectiveness of established regimens, there are no known randomized controlled trials comparing efficacy and safety of DVT prophylaxis methods after PAO. As a result, aspirin remains the most prescribed VTE prophylaxis following PAO. Standard regimens include Aspirin 81 mg or 325 mg, twice daily, per surgeon preference.

#### Heterotopic Ossification Prophylaxis

Heterotopic Ossification (HO) is a common sequela after PAO, with one study of 643 patients undergoing PAO demonstrating a five-year cumulative radiographic probability of 30.4% and symptomatic probability of 3.8% [76]. NSAIDs are an established prophylaxis used to prevent HO, but there is disagreement over the optimal medication, with common regimens including indomethacin or meloxicam for five days [76]. One study of 328 hips in 2021 observed no difference between naproxen and other NSAIDs (meloxicam, indomethacin, celecoxib, aspirin) in HO occurrence [77]. Previous concerns over NSAIDs causing nonunion appear to be unfounded, at least in young patients. A 2022 systematic review involving 238,822 fractures in children revealed a nonunion rate of 0.84%, with no evidence suggesting higher risk of nonunion or delayed bone healing in those treated with NSAIDs [78].

#### Post-Operative Motion and Mobilization

Rehabilitation and physical therapy begin immediately following PAO surgery. In a systematic review of published protocols, 2 out of 3 reporting studies initiated this post-operative mobilization on post-operative day 0 or 1. The same review found that approximately 2/3rds of reporting studies utilized partial weightbearing, and 48% of studies reported the use of crutches as an assistive device [79]. The review was unable to find a consensus on the optimal timing of weightbearing, however, with significant variation in this progression. Multiple published protocols describe limiting active hip flexion, external rotation, and extension past neutral in the immediate postoperative period, though there is no known clinical evidence to support these practices [80, 81] The strongest evidence for this practice comes in the form of expert opinion from a Delphi consensus statement created by 16 physiotherapists deemed experts in PAO rehabilitation. This statement reported that 81% of surveyed therapists agreed on limiting flexion to 90 degrees and external rotation at 90 degrees to 20 degrees for 4–6 weeks postoperatively [82]. Future research is needed to better assess how varying weight bearing restrictions, ROM restrictions, and/or different postoperative physical therapy restrictions affect outcomes and complication rates.

Continuous passive motion (CPM) remains a popular modality to promote initial motion and patient confidence in ROM after surgery, relieve pain, and theoretically prevent adhesion formation. One survey of ANCHOR group surgeons revealed that half prescribed a CPM while inpatient and 6/16 recommended home CPM [16]. Despite this relatively widespread use, there are no randomized trials, cohort studies, or biomechanical studies to suggest clinical benefit from CPM.

### Rehabilitation Phase

The final phase of perioperative care, beginning approximately 6 weeks postoperatively, transitions from range of motion exercises to strengthening exercises, with the eventual goal of returning to pre-operative level of activity and function. The duration of recovery, and the attention to detail needed throughout, should be emphasized to families from the pre-operative period. Importantly, patients must remain engaged with their surgeon and team throughout all phases of care and adherent to postoperative instructions. A recent study on patients undergoing hip arthroscopy showed that at 2-year follow-up, patients who did not attend PT as scheduled, did not adhere to restrictions, or were lost to follow-up had significantly lower patient-reported outcome scores [83]. Similar findings may also apply to recovery from PAO surgery. Exact timelines for weight-bearing status vary between protocols, with many advancing from flat foot weight-bearing to partial progressive weight bearing starting at 6–8 weeks, and full unrestricted weight bearing beginning at 8–12 weeks based upon individual surgeon preferences including time from surgery, radiographic evidence of healing and adequate muscular control. Published evidence for specific rehabilitation protocols and timelines is limited to expert opinion from the Delphi consensus statement from 16 expert PAO physiotherapists. The consensus statement agrees that patients should achieve full hip ROM by 12–16 weeks at maximum, and recommends following specific guidelines for return to sport, with radiographic evidence also strengthening decisions to increase activity or return to sport [82]. A systematic review from 2023 demonstrated that over 70% of competitive athletes are able to return to sport after PAO in a variety of sports, with reported timelines ranging from 8.8 to 12.8 months [84]. Given this extremely limited evidence base, further high-quality research is needed to better investigate and optimize this crucial phase of PAO recovery.

#### Hardware Removal

Multiple studies have examined hardware removal rates after PAO. A retrospective study on 221 patients undergoing PAO for both FAI and DDH from 2008 to 2015 and followed for at least one year found that 13.6% of patients underwent hardware removal. Patients whose hardware was removed were more likely to be younger with lower BMI and less severe radiographic measures of dysplasia. The study also found a trend towards slightly inferior PROMs in patients who had their hardware removed. In a 2024 single-surgeon study of 302 hips, 23.5% of hips underwent hardware removal, and 6.9% of screws broke during removal. Risk of breakage was higher with 3.5‑mm screws and longer time to removal, and lower with 4.5‑mm screws and earlier removal (< 12 months) [85]. While the objective of PAO is to prevent arthritis and later arthroplasty, many patient will require a total hip arthroplasty (THA) at some point [31, 86, 87]. Overall outcomes of THA following PAO are strong, with mid-term follow-up studies reporting improvements in patient reported outcome measures and high survivorship rates [88]. Risk of infection following post-PAO THA remains a concern, however published cohort studies with mid- and long-term follow-up do not demonstrate higher infection rates [88–90]. Hardware removal, whether for THA or for symptomatic hardware, remains controversial and variable between practices. Future research into hardware removal and the influence on THA is necessary.

## Conclusions

PAO has excellent short- and long-term results for patients with symptomatic hip dysplasia. Evidence surrounding outcomes, technical details, and preoperative risk factors for complications is strong. However, limited data exists on many perioperative management practices, including preoperative mental health screening and management, preoperative laboratory evaluation and nutritional optimization, preoperative education, VTE prophylaxis, and postoperative pain control. Differing rehabilitation protocols represents the largest gaps in evidence, with evidence-based changes having the potential for the highest impact on patient outcomes. Strong evidence does exist for control of blood loss intraoperatively and intraoperative pain control methods. In order to optimize post-operative outcomes and to minimize risks for complications, further study is needed to continue to define best practices for perioperative management of PAO.

## Key References

- Clohisy JC, Ackerman J, Baca G, Baty J, Beaulé PE, Kim Y-J, et al. Patient-Reported Outcomes of Periacetabular Osteotomy from the Prospective ANCHOR Cohort Study. J Bone Jt Surg. 2017;99:33–41. 10.2106/JBJS.15.00798.
  - ○ One of the highest‑quality prospective multicenter outcomes dataset in hip preservation, establishing the long‑term patient‑reported benefit of PAO. Importantly, this study identifies patient-specific factors influencing PAO outcomes.
- Willey M, Spiker AM, Schmitz MR, Belzile EL, Sierra RJ, Clohisy J, et al. Peri-Operative Management of Periacetabular Osteotomy: A Report of Current Practices from the Anchor Group, Supporting Literature, and Areas for Future Investigation. Iowa Orthop J. 2024;44:159–66.
  - ○ This study identifies current practices and the variation between centers in PAO perioperative management, and highlights areas where evidence is lacking. By mapping the existing landscape, it directly informs the rationale behind this review and helps define the perioperative domains most in need of standardization.
- Richard HM, Nguyen DC, Podeszwa DA, De La Rocha A, Sucato DJ. Perioperative Interdisciplinary Intervention Contributes to Improved Outcomes of Adolescents Treated With Hip Preservation Surgery. J Pediatr Orthop. 2018;38:254–9. 10.1097/BPO.0000000000000816.
  - ○ This article provides empirical evidence that structured, multidisciplinary perioperative pathways improve outcomes in adolescent hip preservation surgery, supporting the concept that coordinated perioperative management is not just theoretical but outcome modifying. Its conclusions strengthen the argument for systematic perioperative optimization.
- Gornitzky AL, Zaltz I, Hartwell MJ, Bedi A, Kelly BT. The Layer + Model: Incorporating Psychosocial Considerations into Hip Preservation Surgery. Curr Rev Musculoskelet Med. 2025;19:5. 10.1007/s12178-025-09994-3.
  - ○ This reference introduces the most comprehensive conceptual framework integrating psychosocial factors into hip preservation care, emphasizing that perioperative management must extend beyond biomechanics and surgical technique. This framework justifies the inclusion of mental health, expectation setting, and behavioral components in modern PAO perioperative protocols.
- Disantis AE, Ruh E, Martin R, Enseki K, McClincy M. Rehabilitation Guidelines for Use Following a Periacetabular Osteotomy (PAO): A North American Based Delphi Consensus. Int J Sports Phys Ther [Internet]. 2022 [cited 2025 Dec 19];17. 10.26603/001c.38043.
  - ○ The most in-depth consensus based rehabilitation guideline for PAO, this study provides expert derived standards for postoperative precautions, progression, and return to sport criteria. Additionally, the study demonstrates where rehab has been standardized and where institutional variability still exists.

## Data Availability

No datasets were generated or analysed during the current study.
